# Loss of T-cell quiescence by targeting Slfn2 prevents the development and progression of T-ALL

**DOI:** 10.18632/oncotarget.9390

**Published:** 2016-05-17

**Authors:** Aviya Goldshtein, Shani Mistriel Zerbib, Ibrahim Omar, Leonor Cohen-Daniel, Daniel Popkin, Michael Berger

**Affiliations:** ^1^ The Lautenberg Center for Immunology and Cancer Research, The Biomedical Research Institute Israel Canada of the Faculty of Medicine, The Hebrew University Hadassah Medical School Jerusalem, Israel; ^2^ Department of Dermatology, Case Western Reserve University, Cleveland, OH, USA

**Keywords:** T-cell quiescence, T-ALL, Schalfen2, Notch1, p53

## Abstract

T-cell acute lymphoblastic leukemia (T-ALL) is an aggressive malignancy of thymocytes. Despite significant improvement in the treatment of T-ALL, approximately 20% of children and most adults undergo relapse. Previous findings demonstrated that loss of T-cell quiescence due to a mutation in the Slfn2 gene (*elektra*) leads to acquisition of an aberrant developmental program by which T-cells lose their renewal capabilities and undergo apoptosis. Here we show that the *elektra* mutation in Slfn2 completely prevents a severe lymphoproliferative disease caused by overexpression of BCL2 in combination with Fas deficiency in mice. Moreover, Slfn2 impaired-function protects mice from experimental disease similar to human T-ALL by severely impairing the proliferation potential and survival of leukemic T-cells, partially by activation of the p53 tumor suppressor protein. Our study suggest that in certain malignancies, such as T-ALL, a novel therapeutic strategy may be applied by imposing aberrant development of leukemic cells. Furthermore, as the *elektra* mutation in Slfn2 seems to impair only T-cells and monocytes, targeting Slfn2 is expected to be harmless to other cell types, and thereby could be a promising target for treating malignancies. Together our results demonstrate the potential of targeting Slfn2 and its human paralog for T-ALL treatment.

## INTRODUCTION

T-cell acute lymphoblastic leukemia (T-ALL) is an aggressive malignancy of thymocytes. The disease represents 15% of pediatric and 25% of adult acute lymphoblastic leukemia cases [1]. T-ALL is thought to result from malignant thymocytes that arise at defined stages during intrathymic T-cell differentiation [2]. Transformation events occur during crucial steps of thymocyte development that have been related to the expression of certain oncogenes such as TAL1, LMO1/2 and NOTCH1, which have been closely linked to a developmental arrest at a particular stage of normal thymocyte development.

In the hematopoietic system, NOTCH1 is strictly required for the commitment of multipotent hematopoietic progenitors to the T-cell lineage and to support T-cell growth, proliferation and survival at multiple stages of thymocyte development [3, 4]. Under physiological conditions, NOTCH1 is activated by ligand binding, followed by its proteolytic cleavages that liberate the intracellular domain of NOTCH1 (ICN1) in the cytoplasm. ICN1 is then translocated to the nucleus and, in concert with other transcriptional activators, induces the expression of target genes, such as c-Myc, that are involved in important oncogenic pathways [5]. Approximately 55% of human T-ALL patients harbor NOTCH1 activating mutations [1–3]. The vast majority of these mutations occur in two domains: the extracellular domain, which lead to ligand-independent activation of NOTCH1, and/or the intracellular domain in the PEST subdomain, which cause enhanced ICN1 activity by increasing its stability. In addition, hyperactive NOTCH1 mutant alleles as well as ICN1 overexpression, in both human and mice bone marrow (BM) cells, lead to induction of T-ALL [3].

The p53 tumor suppressor is a tightly regulated transcription factor that controls many target genes such as the cell cycle arrest-inducing genes p21 and cyclin G1, the pro-apoptotic genes PUMA and BAX, and its negative regulators MDM2 and MDMX. The p53 protein can initiate different biological processes in normal development as well as in response to stress by inducing apoptosis, cell cycle arrest or senescence [6]. The activation and stabilization of p53 is generally regulated by its inhibition by its major negative regulator, MDM2, with the help of other proteins such as ARF, a small protein that inhibits MDM2 and mediates the induction of p53 [7].

One of the mechanisms underlining the initiation of T-cell lymphoma and leukemia by NOTCH1 is the suppression of p53. NOTCH1 suppresses p53 in lymphomagenesis through repression of the ARF-MDM2-p53 network. Attenuation of NOTCH1 expression resulted in a dramatic increase in p53 levels that led to tumor regression by an apoptotic program. Moreover, activation of the p53 pathway either by ionizing radiation or by treatment with the small molecule therapeutic Nutlin showed that p53 can be activated and cause tumor cell death, even in the presence of sustained NOTCH1 activity [8].

Under normal, non-pathogenic conditions T-cells are kept in a quiescent state, which is characterized by an arrest in the G0 phase of the cell cycle, small cell size and low metabolic activity. Upon microbial invasion, these quiescent T-cells are stimulated to enter into active cell cycle and increase their metabolic activity to acquire effector functions. Recent studies have demonstrated that T-cell quiescence must be actively maintained by the action of molecules that include transcription factors and cell cycle regulators [9, 10] and that T-cell activation involves increased expression not only of genes that promote growth and differentiation, but also of genes that suppress a “quiescence program” [11, 12]. A growing number of transcription factors, including the FoxO family [13–15], Klf2 [16], Tob [17], and most recently Tsc1 [18] and Foxp1 [19], have been linked to the regulation of quiescence in immune cells.

The Schlafen (Slfn) genes were first described in mice as a family of genes that are transcribed during thymocyte maturation [20]. This gene family consists of 8 genes in mouse, and 6 genes in human, all residing on the same chromosome adjacent to each other. In mouse, the Slfn gene family codes for three subpopulations of proteins categorized by their length: short (Slfn1 and 2), intermediate (Slfn3 and 4) and long (Slfn5, 8, 9, and 10). All Slfn proteins share a common core region containing a divergent AAA domain (ATPase Associated with various cellular Activities; AAA4), which presumably has ATP-binding activity. The long Slfn proteins also contain a motif similar to the superfamily I helicases [21]. Beside this shared motif, Slfn proteins harbor no sequence similarity to other proteins. Several studies support a role for Slfn members in the immune response. Slfn genes are expressed in immune system tissues and their expression levels vary during T-cell and macrophage development, as well as in response to infections [20–23]. Both Slfn1 and Slfn8 transgenic mice show a reduction in thymus size and thymocyte proliferation relative to wild-type mice [20, 21]. These and other findings, especially from the ectopic expression of Slfn1 in mouse fibroblasts, suggest a role for Slfn proteins in inhibition of cell growth [20, 24]. However, this anti-proliferative activity is not shared by all Slfn genes [21, 25] and has not been confirmed by other studies. [26] Knockout studies of Slfn1 [20] and Slfn3 [27] showed no apparent phenotype, suggesting functional redundancy among Slfn family members.

We had previously described a chemically induced mutation, *Elektra,* in the Slfn2 gene demonstrated that Slfn2 acts as quiescence regulator that is essential for immune defense. Elektra T-cells fail to maintain cellular quiescence and as a consequence, enter a post-mitotic phase, similar to T-cells with a “recently” activated phenotype. In this phase T-cells lose their proliferation potential and undergo cell death in response to proliferation/activation signals, leading to diminished numbers of T-cells in the elektra mutant mice [27].

Here we examined the possibility that inhibition of T-cell quiescence through impairing function of *Slfn2* can reduce and even prevent the development of T-cell leukemia/lymphoma by driving the leukemic cells into post-mitotic phase and thereby preventing their ability to proliferate. We demonstrate that Slfn2 is critical in the pathogenesis of T-ALL induced by ICN1 and that downregulating Slfn2 attenuates the development and the progression of this disease. In addition, we show that the p53 tumor suppressor is involved in the apoptotic death of Slfn2-deficient T-cells, suggesting p53 activation as one of the mechanisms of T-ALL inhibition by downregulation of Slfn2. Overall, our study suggests that targeting Slfn2 holds the potential to constitute a completely novel and ground-breaking strategy for treating T-ALL.

## RESULTS

### The elektra mutation in Slfn2 prevents lymphoproliferative disease mediated by the Bcl2 transgene combined with Fas loss-of-function

Elektra mice overexpressing Bcl-2 in the T-cell compartment, *BCL2(Tg)/Slfn2^eka/eka^*shown to have normal counts of T-cells. In addition, Bcl2 found to be downregulated in elektra T-cells. These results demonstrated that *elektra* T-cells undergo apoptosis *via* the intrinsic apoptotic pathway [27].

**Figure 1:**
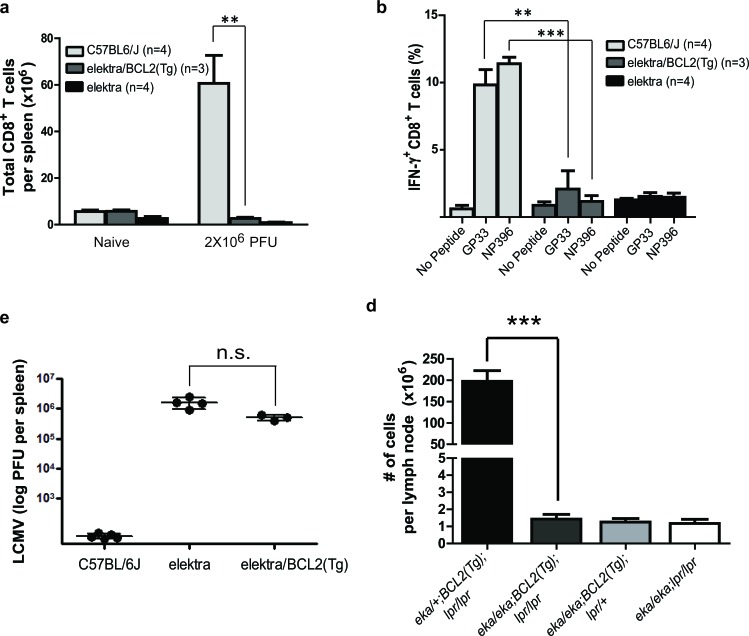
*Elektra* mutation in Slfn2 prevents lymphoproliferative disease mediated by BCL2-transgene combined with FAS loss-of-function **a.** Total CD8+ splenocytes isolated from wild-type, elektra and elektra/BCL2(Tg) mice 7 days after intravenous injection of 2 × 10^6^ PFU of LCMV (Armstrong strain). **b.** Frequency of cells with intracellular IFN-γ expression among CD8^+^ splenocytes re-stimulated *ex vivo* with GP33 or NP396 (peptides derived from LCMV) in the presence of Brefeldin A. **c.** Viral load in spleens of wild-type, elektra and elektra/BCL2(Tg) mice 7 days after intravenous injection of 2×10^6^ PFU of LCMV (Armstrong strain). Each symbol represents an individual mouse; small horizontal lines indicate the mean. n.s., not significant (*P* = 0.0355; two-tailed Student's *t*-test). **d.** The total number of cells from spleens and lymph nodes from 5-month-old littermate offspring from crosses of elektra/elektra and elektra/+ mice expressing a BCL2 transgene (BCL2(Tg)) to C57BL/6J/lpr mice to generate elektra homozygotes overexpressing Bcl-2 in T-cells and lpr mice (*n* = 5 for each strain).

Next, we tested whether blocking the intrinsic apoptotic pathway by overexpression of the BCL2 gene in the T-cell compartment can also restore *elektra* T-cell function *in vivo*. To this end, *BCL2(Tg)/Slfn2^eka/eka^* were subjected to lymphocytic choriomeningitis virus (LCMV- Armstrong strain) infection that its control is mainly dependent on CD8^+^ T-cell. Similar to elektra mice, *BCL2(Tg)/Slfn2^eka/eka^* mice had fewer CD8^+^ T-cells after LCMV infection (Figure 1a). In addition, *ex vivo* re-stimulation of splenocytes from LCMV-infected *BCL2(Tg)/Slfn2^eka/eka^* mice with LCMV-derived peptides (representing immunodominant epitopes of both envelope and nuclear protein antigens) led to significantly fewer IFN-γ-producing CD8^+^ cells than wild-type mice (Figure 1b). Consistent with these results, *BCL2(Tg)/Slfn2^eka/eka^* mice failed to clear LCMV infection similar to elektra mice (Figure 1c). These results demonstrate that even when the propensity for apoptosis is blocked in elektra T-cells, their proliferation capacity is not fully reconstituted. The disruption of both extrinsic and intrinsic apoptotic pathways by combining *BCL2(Tg)* with the *lpr* mutation within the *Fas* gene, respectively, leads to enhanced lymphoproliferative abnormalities as compared to mice with a deficiency in only one pathway [28]. In fact, *BCL2(Tg)/lpr*-transgenic mice exhibit substantially enhanced lymphadenopathy compared with *lpr* or *BCL2(Tg)* only mice, which is mainly explained by the enhanced accumulation of both immature double negative (CD4^−^/CD8^−^) and double positive (CD4^+^/CD8^+^) T-cells [28]. Our results suggest that the *elektra* mutation diminishes the proliferation advantage of *BCL2(Tg)* T-cells. In addition, as we previously showed, *elektra* mutation in Slfn2 completely blocks the enhanced proliferation of *lpr* T-cells [27]. Therefore, we next tested whether the *elektra* mutation is also sufficient to prevent lymphoproliferative disease mediated by the Bcl2 overexpression combined with Fas loss-of-function. To perform this experiment, we generated *BCL2(Tg)/Fas^lpr/lpr^/Slfn2^eka/eka^* mice and determined their propensity to develop lymphoproliferative disease. While *BCL2(Tg)/Fas^Ipr/lpr^/Slfn2^wt/eka^* mice showed enhanced lymphadenopathy and had a significantly larger number of cells in lymph nodes compared with control littermates that had an intact Fas (*BCL2(Tg)/Fas^wt/lpr^/Slfn2^wt/eka^*) none of the abnormalities were observed in *BCL2(Tg)/Fas^Ipr/lpr^/Slfn2^eka/eka^* mice (Figure 1d), suggesting that even T-cells lacking the two main apoptotic pathways dependent on BCL2 and FAS, must have an intact Slfn2 gene to support T-cell proliferation, immortalization and subsequent development of lymphadenopathy, thereby implying that Slfn2 may have a role in T-cell malignancies such as T-ALL.

### The *elektra* mutation in Slfn2 significantly protects mice from experimental disease similar to T-ALL

**Figure 2:**
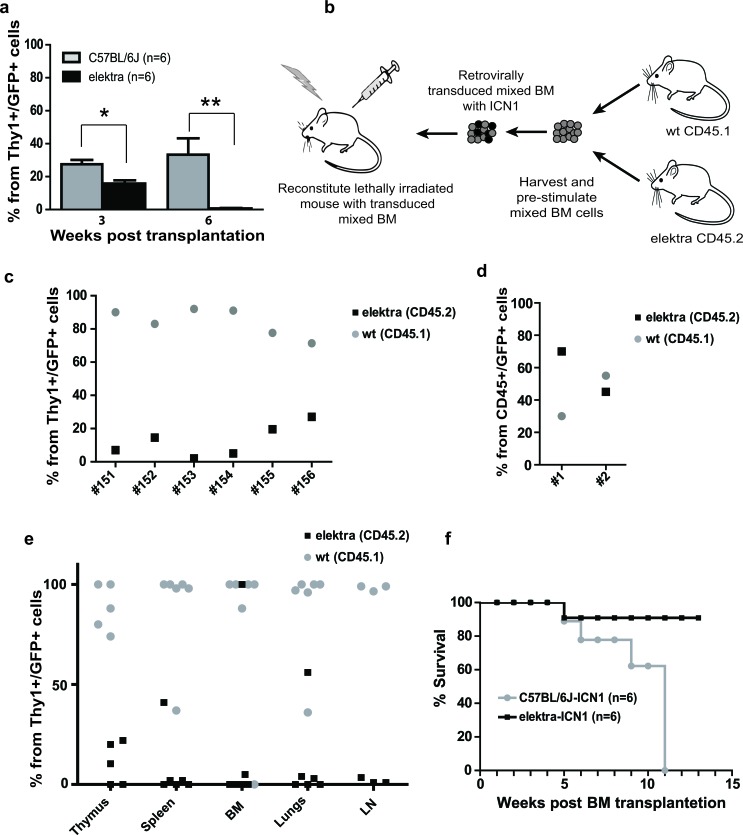
*Elektra* mutation in Slfn2 inhibits development and progression of T-ALL induced by ICN1 **a.** Percentages of leukemic DP and DN T-cells in the blood of recipient mice reconstituted with either elektra (black) or wild-type (grey) BM cells transduced with retrovirus expressing human intracellular NOTCH1 (ICN1) 3 and 6 weeks after transplantation (*n* = 6). **b.** Schematic illustration of the mixed BM chimera experiments. Equal numbers of BM cells from 5FU-treated CD45.1 wild-type mice and BM cells from CD45.2 syngeneic elektra mice were mixed and transduced with retroviruses expressing ICN1/GFP or control retroviruses expressing only GFP (empty MIGR1). The transduced mixed BM cells were then transplanted into irradiated recipient mice. **c.** Percentages of Thy1+ GFP+ cells in the blood of recipient mice reconstituted with elektra CD45.2 (black) together with wild-type CD45.1 (grey) BM cells transduced with retrovirus expressing ICN1 (#151-156) (ratio 1:1) 4 weeks after transplantation. **d.** Percentages of CD45+ GFP+ cells in the blood of recipient mice reconstituted with elektra CD45.2 (black) together with wild-type CD45.1 (grey) BM cells transduced with empty MIGR1 retrovirus (ratio 1:1) 4 weeks after transplantation. **e.** Percentages of Thy1+ GFP+ cells in different tissues of recipient mice transplanted with elektra CD45.2 (black) together with wild-type CD45.1 (grey) BM cells transduced with retrovirus expressing ICN1 (ratio 1:1) 7 weeks after transplantation. **f.** Survival curves of recipient mice reconstituted with either elektra (black) or wild-type (grey) BM cells transduced with retrovirus expressing human intracellular ICN1 (*n* = 6).

To test the role of Slfn2 in T-cell malignancies, we analyzed whether *Slfn2* impaired-function in elektra mice can inhibit the development of T-ALL in an established animal model for T-ALL involving the expression of oncogenic NOTCH1 (intracellular NOTCH1 fragment, ICN1). This model entails the transplantation of wild-type or elektra mutant hematopoietic progenitors carrying ICN1 introduced by retroviral transduction (WT^ICN1^ or elektra^ICN1^, respectively) into irradiated recipient wild-type mice [29, 30]. Recipient mice transplanted with either wild-type or elektra ICN1-transduced BM cells were observed to have immature double positive and double negative T-cells in their blood as early as three weeks from transplantation, with slightly fewer such immature T-cells in the blood of elektra^ICN1^ transplanted mice (Figure 2a). However, while immature double positive and double negative T-cell numbers continued to increase in mice that received wild-type cells, mice transplanted with elektra cells showed no trace of these immature T-cells in their blood (Figure 2a). In addition, in a mixed BM chimera setting in which irradiated mice were transplanted with a mixture (1:1 ratio) of ICN1 retrovirus transduced wild-type (CD45.1) and elektra (CD45.2) BM cells (Figure 2b), a highly significant overtake of wild-type origin immature double positive and double negative T-cells was observed in the blood already 4 weeks after transplantation (Figure 2c) and in the BM, thymus, lungs, lymph nodes and spleen 7 weeks after transplantation (Figure 2e). In contrast, when control virus was used in the same setting, no remarkable difference was observed with regard to the ratio between wild-type and elektra cells, suggesting that the elektra mutation does not impair the transplantation capacity of hematopoietic stem cells (Figure 2d). These results suggest that the elektra mutation severely impairs the proliferation potential that ICN1 provides to immature T-cells. Most importantly, in line with these findings, most of the *elektra^ICN1^* transplanted mice were protected from ICN1-induced mortality (Figure 2f), indicating that the *elektra* mutation in Slfn2 significantly protects the mice from experimental disease reminiscent of human T-ALL. These results suggest that Slfn2 impaired-function can avert the growth of pre-leukemic T-cells even in the presence of continued Notch1 activity.

### P53 loss-of-function increases *elektra* T-cell survival but fail to restore their proliferation capacity

Next we aimed to understand the mechanism governing the growth suppression of the ICN1 induced T-ALL mediated by the *elektra* mutation in the Slfn2 gene.

**Figure 3:**
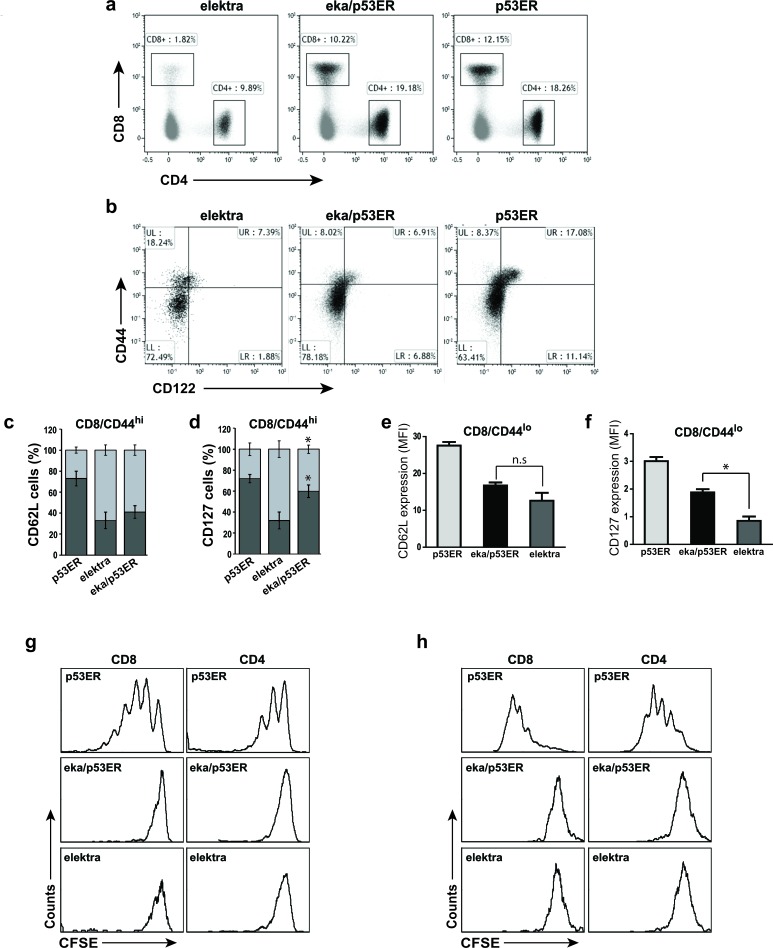
Elektra/p53ER^**TAM**^ mice have normal T-cell numbers but cells still lack the ability to proliferate **a.** Flow cytometry analysis of the expression of CD4 and CD8 in cells from the spleen of elektra, elektra/p53ER^TAM^ and p53ER^TAM^ mice. **b.** Flow cytometry analysis of the staining of CD44 and CD122 (IL-2Rβ) in splenic CD8^+^ cells from p53ER^TAM^, elektra/p53ER^TAM^ and elektra mice. **c.**, **d.** Frequency of CD62L^hi^ and CD62L^lo^ cells in the CD44^hi^ population of CD8^+^ T-cells from p53ER^TAM^, elektra/p53ER^TAM^ and elektra mice (*n* = 4 per genotype). **e.**, **f.** Mean fluorescence intensity (MFI) of **e.** CD62L (L-selectin) and **f.** CD127 (IL7Rα) staining in the CD44^lo^ population of CD8^+^ T-cells from p53ER^TAM^, elektra/p53ER^TAM^ and elektra mice (*n* = 4 per genotype). *P < 0.001 (two-tailed Student's *t*-test). Results are representative of two experiments. Error bars, s.e. **g.** CFSE intensity of CD4^+^ (right panels) and CD8^+^ (left panels) T-cells from spleens of immune-depleted mice 7 days post-adoptive transfer of CFSE-labeled splenocytes from p53ER^TAM^, elektra/p53ER^TAM^ and elektra mice.**h.** CFSE intensity of splenic CD4^+^ (right panels) and CD8^+^(left panels) T-cells stimulated for 72 h with anti-CD3ε plus anti-CD28. Results are representative of three experiments.

The suppression of the p53 tumor suppressor was shown to be crucial to the initiation of T-cell lymphoma and leukemia by NOTCH1 [8]. NOTCH1 suppresses p53 in lymphomagenesis through repression of the ARF-MDM2-p53 network. Activation of the p53 pathway can be stimulated and lead to tumor cell death, even in the presence of sustained NOTCH1 activity [8]. These findings suggest that one of the mechanisms by which downregulation of Slfn2 attenuates T-ALL development and progression is by activation of the p53 pathway. To test this hypothesis, we examined whether elektra T-cell death is indeed mediated by p53 by evaluating whether p53 downregulation can rescue the elektra T-cell phenotype. To this end, p53 knock-in mice, *p53ER^TAM^*, where p53 is inactive under standard conditions (as long as Tamoxifen wasn't injected to the mice) [31], were crossed to *elektra* mice to generate *p53ER^TAM^/Slfn2^eka/eka^* double transgenic mice. T-cells from these mice were subjected to immunophenotyping, *in-vivo* expansion assay and *ex vivo* activation assay. The percentages of CD4^+^ and CD8^+^ T-cells from spleens of *p53ER^TAM^/Slfn2^eka/eka^* mice were similar to those from *p53ER^TAM^* mice (Figure 3a). However, similar to elektra T-cells, *p53ER^TAM^/Slfn2^eka/eka^* CD44^hi^ CD8^+^ T-cell population failed to gain a memory-like phenotype of CD44^hi^/CD122^+^, and were mostly CD122^−^ (Figure 3b). Most of this CD44^hi^CD122^−^ population showed a complete shedding of CD62L (L-selectin) (Figure 3c and supplementary Figure 1a) and only partial restoration of IL-7 receptor α-chain (IL-7Rα (CD127)) expression (Figure 3d and supplementary Figure 1b). In addition, the CD44^lo^ (naive) population of CD8^+^ and CD4^+^ T-cells from *p53ER^TAM^/Slfn2^eka/eka^* mice had low expression of both CD62L (Figure 3e and supplementary Figure 1c) and IL-7Rα (Figure 3f and supplementary Figure 1d) similar to elektra T-cells. These results suggest that although p53 deficiency restores the elektra T-cells number, it fails to repair their capability to maintain quiescence as well as to acquire a memory like phenotype.

To further examine the impact of p53 activity on the proliferative capacity of elektra T-cells, we examined the *p53ER^TAM^/Slfn2^eka/eka^* T-cell response to homeostatic expansion signals. *p53ER^TAM^/Slfn2^eka/eka^*, *p53ER^TAM^* and *elektra* (CD45.2) splenocytes were labeled with the cytosolic dye CFSE and adoptively transferred into sublethally irradiated wild-type (CD45.1) recipients. As expected, 7 days after transfer, *p53ER^TAM^* T-cells underwent proliferation and elektra T-cells failed to proliferate and were barely detectable (Figure 3g). *p53ER^TAM^/Slfn2^eka/eka^* T-cells, although detectable, did not proliferate (Figure 3g). Similar results were observed with *p53ER^TAM^/Slfn2^eka/eka^* T-cells in an *ex vivo* activation assay (Figure 3h).

Together these results suggest that *elektra* T-cell death is mediated by p53. However, the developmental and proliferation defects of *elektra* T-cells cannot be attributed to p53 and most likely are related to other factors, which still need to be explored.

### Attenuation of T-ALL development by the elektra mutation in Slfn2 is partially mediated by the activation of the p53 pathway

**Figure 4:**
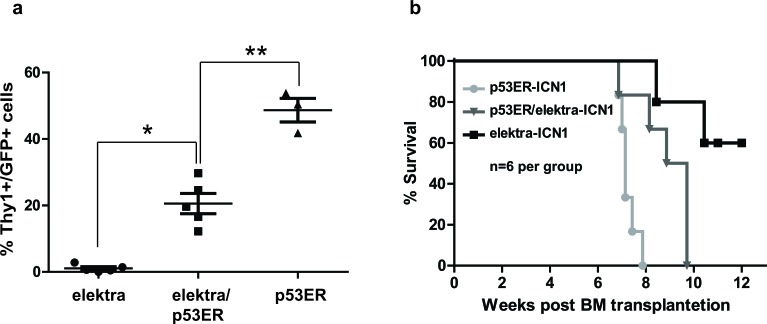
Down regulation of p53 partially restores development and progression of T-ALL in elektra mice **a.** Percentages of leukemic DP and DN T-cells in the blood of recipient mice reconstituted with either, elektra, elektra/p53ER or p53ER BM cells transduced with retrovirus expressing human intracellular NOTCH1 (ICN1) 4 weeks after transplantation. **b.** Survival curves of recipient mice reconstituted with either, elektra (light grey), elektra/p53ER (grey) or p53ER(black).(*n* = 6).

Next, to directly evaluate whether the *elektra* mutation in Slfn2 attenuates T-ALL development and progression is by activation of the p53 pathway, we subjected the *p53ER^TAM^/Slfn2^eka/eka^* double transgenic mice to the ICN1 induced T-ALL model. Elektra, *p53ER^TAM^/Slfn2^eka/eka^* and *p53ER^TAM^/Slfn2^wt/wt^* hematopoietic progenitor cells carrying ICN1 introduced by retroviral transduction were transplanted into irradiated recipient wild-type mice. Six weeks after transplantation, recipient mice transplanted with *p53ER^TAM^/Slfn2^eka/eka^* ICN1-transduced BM cells showed to have significantly higher percentages of T-ALL cells in the blood comparing to the recipient mice transplanted with elektra ICN1-transduced BM cells and reduced percentages of T-ALL cells in the blood comparing to the recipient mice transplanted with *p53ER^TAM^/Slfn2^wt/wt^* ICN1-transduced BM cells (Figure 4a).

Moreover, in line with these findings, the survival of *p53ER^TAM^/Slfn2^eka/eka^*-ICN1 transplanted mice was severely reduced as compare to the elektra^ICN1^ transplanted mice, yet increased as compare to the *p53ER^TAM^/Slfn2^wt/wt^*-ICN1 transplanted mice (Figure 4b). These results indicate that the *elektra* mutation in Slfn2 significantly protects the mice from ICN1-induced T-ALL is partially mediated by the activation of the p53 pathway.

### Downregulation of Slfn2 attenuates preexisting T-cell lymphoma

**Figure 5:**
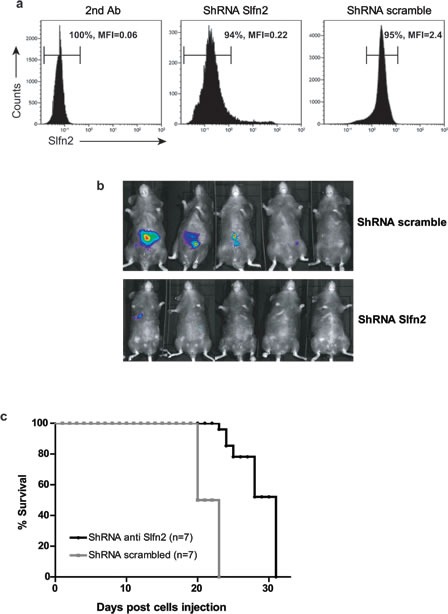
Downregulation of Slfn2 attenuates preexisting T-cell lymphoma **a.** Flow cytometry analysis of Slfn2 expression in the EL4 lymphoma cell line stained with only secondary antibody (left panel), transduced with shSlfn2 (middle panel) or transduced with non-specific scrambled shRNA (right panel). **b.** Analysis of T-cell lymphoma development in Rag1^−/−^ mice by the IVIS Kinetic Bioluminescent Fast Imaging system 13 days after EL4 cell line injection. Upper panel: Rag1^−/−^ mice injected with EL4 cells that were previously transduced with non-specific scrambled shRNA lentivirus together with lentivirus expressing the luciferase reporter gene. Lower panel: Rag1^−/−^ mice injected with EL4 cells that were previously transduced with Slfn2 shRNA together with lentivirus expressing the luciferase reporter gene. **c.** Survival curves of Rag1^−/−^ mice injected with EL4 cells transduced with either Slfn2 shRNA (black) or non-specific scrambled shRNA (grey) lentivirus (*n* = 7).

Our results demonstrate that Slfn2 deficiency leads to the attenuation of T-ALL development. To evaluate the therapeutic potential of targeting Slfn2 we next tested whether downregulation of Slfn2 could also decrease the proliferation and survival of already existing malignant T-cells. To test this possibility we used shRNA to downregulate Slfn2 expression in EL4, a murine T-cell lymphoma line. A series of shRNAs targeting murine Slfn2 were designed and tested for their ability to reduce Slfn2 protein expression by flow cytometry analysis (data not shown). The most effective shRNA was shSlfn2-4, which reduced Slfn2 expression by approximately 10-fold in 94% of the cells (Figure 5a). EL4 cells were transduced with lentivirus expressing shSlfn2-4 or nonspecific scrambled shRNA together with lentivirus expressing the luciferase reporter gene and injected into Rag1^−/−^ lymphocyte-deficient mice. The progression in EL4 proliferation was measured using the IVIS Spectrum *in vivo* imaging system 13 days post-injection. Rag1^−/−^ mice injected with shSlfn2-4-transduced EL4 cells showed reduced luciferase activity 13 days post-injection (Figure 5b). Moreover, Slfn2 downregulation prolonged survival with a median survival of 31 days compared with mice that received EL4 cells transduced with nonspecific scrambled shRNA with a median survival of 23 days (Figure 5c). These results demonstrate that targeting Slfn2 in T-cell lymphoma impairs the potential of these cells to establish an active disease in mice and that Slfn2 controls the proliferation capacity of the tumor cells, thus when it is downregulated mice survive for a longer period.

### Slfn2 attenuates T-cell lymphoma through p53 activation

**Figure 6:**
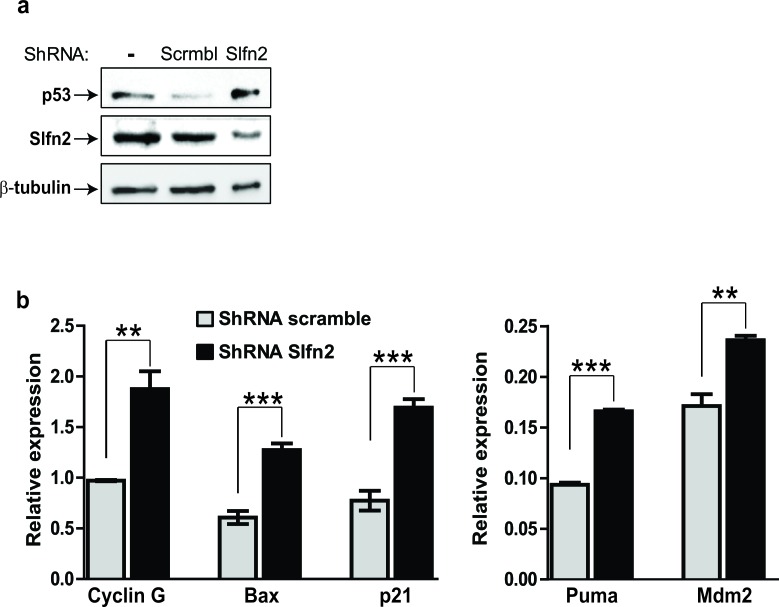
Downregulation of Slfn2 activates the p53 tumor suppressor in T-cell lymphoma **a.** Immunoblot analysis of p53 and Slfn2 in EL4-lymphoma cell line, un-transduced or transduced with shSlfn2 or non-specific scrambled shRNA expressing lentiviruses.α-tubulin expression is shown as a loading control. **b.** Real-time PCR analysis of p53 target genes (indicated in the figure) in EL4-lymphoma cell line transduced with shSlfn2 (black bar) or non-specific scrambled shRNA (grey bar) expressing lentiviruses.

The finding that the elektra mutation in Slfn2 leads to the suppression of ICN1-induced T-ALL can be significantly abrogated by p53 deficiency together with the finding that downregulation of Slfn2 in EL4 cells leads to their growth inhibition led us to postulate that downregulation of Slfn2 in EL4 cells will lead to the activation of the p53 tumor suppressor in these cells. To test this notion we first sequenced the TP53 gene from EL4 cells and found it to be intact (supplementary Figure 2). Then we tested the activation of p53 in response to downregulation of Slfn2. The p53 protein levels were increased in EL4 cells expressing shRNA for Slfn2 as compare to cells expression scrambled shRNA (Figure 6a). In line with this result, p53 target genes were also elevated in Slfn2-downregulated EL4 cells (Figure 6b).

## DISCUSSION

The prognosis of T-ALL has gradually improved over recent years with the introduction of intensified chemotherapy, with cure rates in modern protocols reaching over 75% in children and about 50% in adults [32]. However, the outcome of T-ALL patients with primary resistant or relapsed leukemia remains poor [32, 33]. Therefore, current research efforts are focused on the search for more effective and less toxic anti-leukemic drugs [34]. In this work we present a novel approach for T-ALL treatment by targeting T-cell quiescence. We demonstrated that mice with the mutated allele of the Slfn2 gene *elektra* did not develop a lymph-proliferative disease although the mice lack the two main apoptotic pathways. Moreover, in an ICN1-induced T-ALL model, *elektra* T-ALL cells could not proliferate and survive compared with wild-type T-ALL cells. In line with these results, recipient mice that were transplanted with elektra ICN1-transduced BM had significantly higher survival rates than recipient mice that were transplanted with wild-type ICN1-transduced BM. Additionally, the BM chimera experiment enabled us to test the proliferation capacity of *elektra* and wild-type T-ALL cells in similar settings, thereby neutralizing the transduction and cell culturing effects. When a control virus was used in the same setting, no significant difference was observed with regard to the ratio between wild-type and *elektra* cells. This is an important result showing that the *elektra* mutation does not impair the transplantation capacity of hematopoietic stem cells, and that the cells can populate an immune-depleted mouse at normal efficiency.

Our results are surprising; although the elektra phenotype was shown not to effect thymus development [27], still, T-ALL a malignancy which is considered to developed from thymocytes, could not progress in the absence of intact Slfn2. These results led us to the conclusion that Slfn2 is essential for T-ALL cell survival and proliferation.

Inactivating mutations in the p53 tumor suppressor gene are associated with poor prognosis in different types of leukemia and lymphoma, as well as in T-ALL [8, 35, 36]. Moreover, activation of p53-signaling pathways with specific drugs induces cell cycle arrest and apoptosis in childhood acute lymphoblastic leukemia (ALL) [37]. To elucidate the mechanism underlying the suppression of T-ALL in elektra mice, we examined the p53 pathway. We demonstrated a rescue of both CD8^+^ and CD4^+^ T-cell numbers in the *p53ER^TAM^/Slfn2^eka/eka^* double mutant mouse. Our results clearly demonstrate the involvement of p53 tumor suppressor in elektra T-cell death. However, while p53 inactivation restored the number of elektra T-cells, it had no impact on the semi-activated phenotype and impaired proliferation of elektra T-cells, as we demonstrated in both *in vivo* proliferation and *ex vivo* activation assays. These results support the idea that the elektra phenotype entails both impaired ability to maintain quiescence and fragility upon activation/proliferation stimulus. P53 seems to be involved in the increased fragility after activation/proliferation of elektra T-cells, but not in their impaired enforcement of quiescence. Therefore, stress signals may be induced in elektra T-cells due to their inability to maintain quiescence; this may promote p53 activation, which in turns induces apoptosis by transcriptionally activating its target genes.

The ability to suppress the progression of NOTCH1-induced lymphomagenesis by p53 activation was demonstrated previously [8, 38]. These studies showed that activation of p53 in T-ALL cells causes tumor cell death, even in the presence of sustained NOTCH1 activity. This reinforced the idea that the activation of the p53 pathway is involved in the elektra-suppression of ICN1-induced T-ALL. Indeed, we could clearly show that p53 deficiency partially restored the ability of ICN1 to induce T-ALL in elektra BM cells. In addition, our results demonstrate that p53 is activated upon downregulation of Slfn2 and induce their cell death. However, our results also suggest that there are additional players other than p53 that display an important role in the effect of targeting Slfn2 on the development and progression of T-ALL. As mentioned above, p53 inactivation restored elektra T-cell number but was unable to rescue T-cell proliferation ability. Moreover, p53 inactivation effects on the progression of *elektra* T-ALL cells and Slfn2-downregulated EL4 cells were only partial. Therefore, even if p53 is downregulated in T-ALL cells (a common event in T-ALL relapses), targeting Slfn2 is still expected to remain effective.

The study presented herein is different from previously proposed therapeutic strategies, which attempted to block cell cycle progression or directly induce apoptosis of leukemic cells. We suggest that in some instances, such as in the case of T-ALL, a reverse therapeutic strategy may be applied by promoting aberrant development of leukemic cells. The *elektra* mutation in Slfn2 seems to impair only T-cells and monocytes [27]. Therefore, targeting Slfn2 is expected to be harmless in other cell types and tissues. Importantly, Slfn2 has no oncogenic properties; its overexpression appears to lead to cell growth arrest [20, 24] and therefore does not seem to hold the potential to cause cancer. Yet, its wild-type function seems to be critical for the establishment of T-ALL. Thus, targeting Slfn2 or its pathway holds the potential to constitute a completely novel strategy for treating T-ALL. To support this notion, we found that targeting Slfn2 by shRNA delayed lymphoma progression in Rag1^−/−^ mice and prolonged their survival. These results imply that Slfn2 and its human paralogs might be legitimate candidates as targets to treat lymphoproliferative diseases.

In conclusion, our study demonstrates for the first time that targeting Slfn2 holds the potential to constitute a novel strategy for treating T-ALL.

## MATERIALS AND METHODS

### Mice

Elektra mice were previously generated as described in Berger et al. [27] Animals were maintained in a specific pathogen-free environment. The *p53ER*^TAM^mice were generously donated by Professor Gerard Evan. C57BL/6J (wild-type), B6.Cg-Tg(BCL2)25Wehi/J, B6.MRL-Faslpr/J, B6.129S7-Rag1^tm1Mom^/J (Rag1^−/−^) and C57BL/6.SJL (PtprcaPep3b; Ly5.1) (CD45.1) mice were from The Jackson Laboratory.

### BM transplantation

Donor mice were IP injected with 150 mg/kg 5-fluorouracil (5-FU) five days before cell harvesting. BM cells were extracted as previously described. [39] Cells were then transduced with retroviruses expressing ICN1/GFP or only GFP and incubated for 2 days at 37°C. 24 h before transplantation, recipient mice were irradiated (700 rad) using an XRAD-225 machine. The irradiated recipients were then divided into two groups that were injected with 2.5×10^6^ BM cells from either elektra donor mice or C57BL/6J donor mice by tail vein injection. Mice were maintained for 4 weeks post-irradiation on water containing neomycin. For BM chimera experiments, equal amounts of elektra and wild-type BM cells were mixed before retrovirus transduction.

### Cytokines

The following cytokines were used for BM culture: IL3, IL6 (20 ng/ml), stem cell factor (50 ng/ml), and TPO (100 ng/ml) (all from Peprotech).

### Viral constructs

All gene silencing was performed using the pLKO.1-puro lentiviral vector targeting sequences purchased from Sigma. ICN1_ MigR1 and MigR1 vectors were obtained from Professor Yafenof's laboratory.

### Retrovirus generation and BM infection

For retrovirus production, HEK-293T cells were co-transfected with the viral backbone vector, pCMV-VSVG and pCL-Eco packaging vectors using Mirus TransIT-293 transfection reagent. Virus-containing supernatants were collected 48 and 72 h post-transfection following their concentration in 70000 x *g* at 4°C using an ultracentrifuge. Infected BM cells were incubated with concentrated viral supernatants in the presence of 6 μg/ml polybrene (Sigma) for 4 h in bacterial tubes and then transferred back to 6-well plates for another 2 days.

### Flow cytometry detection of T-ALL population

Blood was collected from recipient mice 3 weeks post-BM transplantation. Red blood cells were removed from the samples by erythrocytes lysis buffer containing NH_4_Cl, KHCO_3_ and EDTA 0.5 M. The remaining cells were then re-suspended in the relevant antibody mixture diluted in PBS containing 2% FBS. Cells were then analyzed using a Gallios flow cytometer (Beckman Coulter). Analysis was performed by Kaluza software.

### Adoptive transfer of T-cells

A total of 1×10^7^ CFSE-labeled spleen cells were IP injected into C57BL/6J recipient mice that had been sublethally irradiated (600 rads) 24 h earlier. 7 days after adoptive transfer, spleen cells were harvested, stained for CD45.1, CD8 and CD4 and analyzed by flow-cytometry for CFSE dilution.

### T-cell proliferation assay

A total of 3 × 10^5^ CFSE-labeled spleen cells were activated in 96-flat-well plates by anti-CD3 (0.2 μg/ml), anti-CD28 (0.2 μg/ml) and IL-2 (100 ng/ml). The cells were analyzed 72 h after activationby flow cytometry as described in the adoptive transfer section.

### Lentivirus generation and EL4 infection

For lentivirus production, 293T cells were co-transfected with the viral backbone vector, PMDG and gag-POL packaging vectors donated from Professor Ben-Neriyha's lab using Mirus-TransIT-293 transfection reagent according to standard protocol. EL4 cells were transduced with 5 ml lentivirus in the presence of (6 μg/ml) polybrene. Two days post-infection, puromycin (2.5 μg/ml) was added to the cells for another 1 day at 37°C for the selection of cells containing Slfn2 shRNA or scrambled shRNA. After selection, the cells were infected with the lentivirus containing luciferase donated from Dr. Granot's lab. A total of 6 × 10^5^ infected cells were injected IP into Rag1^−/−^ mice. The mice were monitored for lymphoma development by the IVIS Kinetic (Caliper Life Sciences) Bioluminescent Fast Imaging system at 13 and 20 days post-injection. Before imaging, the mice were injected with luciferin at 3 mg per mouse (Megapharm, catalog number: 82250).

### Antibodies

The following antibodies were used: CD90.2 (Thy 1.2) (53-2.1), CD62L (MEL-14), CD45.1 (A20), CD122 (IL2RB) (5H4), CD127 (IL7R) (SB/199), CD8 (RPA-T8, 53-6.7), CD4 (RM4-5), LY6C (HK1.4), CD45.2 (104), CD3 (17A2), TCRB (H57-597), CD44 (IM7), streptavidin goat anti-rabbit (A21244; all from Biolegend) and Slfn2 (Rabbit polyclonal antibody made by Covance for our laboratory).

## SUPPLEMENTARY MATERIAL FIGURES


