# Supplementary material for: Loss of T-cell quiescence by targeting Slfn2 prevents the development and progression of T-ALL
Source: Oncotarget. 2016 May 17;7(30):46835–47. doi: 10.18632/oncotarget.9390 (PMC5216906; doi:10.18632/oncotarget.9390)
Supplement: Supplementary file 1 [file oncotarget-07-46835-s001.pdf]

“Loss of T-cell quiescence by targeting Slfn2 prevents the development and progression of T-ALL”

Supplementary Material

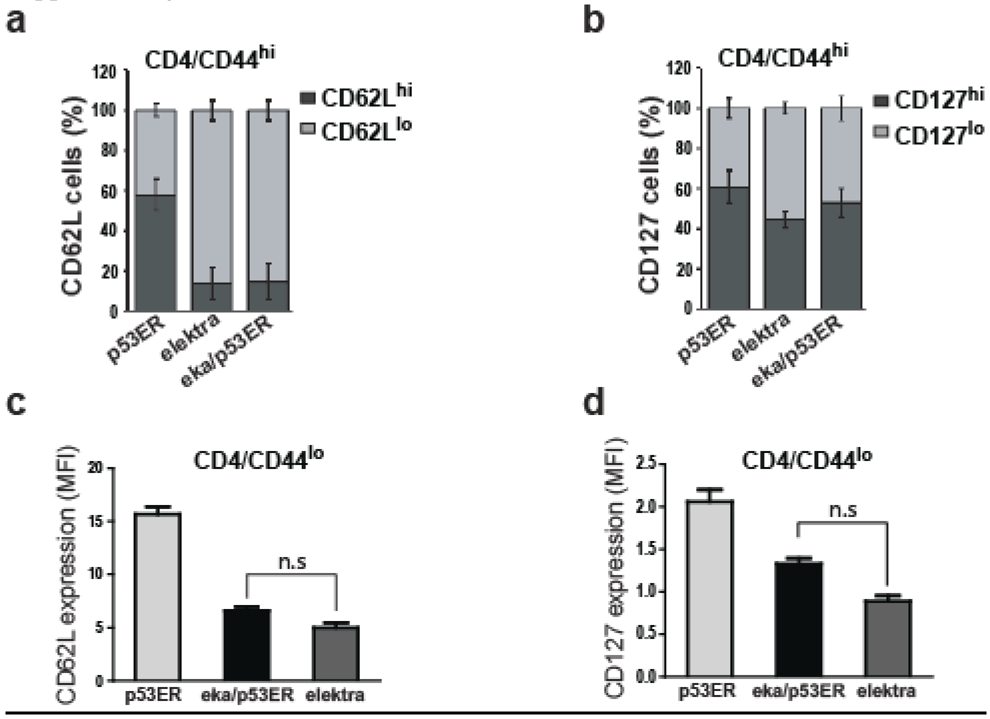

Supplementary Figure 1: Elektra/p53ER^{TAM} CD4^{+} T-cells exist in a semiactivated state. **(a)** Frequency of CD62L^{hi} and CD62L^{lo} cells in the CD44^{hi} population of CD4^{+} T-cells from p53ER^{TAM}, elektra/p53ER^{TAM} and elektra mice (n=4 per genotype) **(b)** Frequency of CD127^{hi} and CD127^{lo} cells in the CD44^{hi} population of CD4^{+} T-cells from p53ER^{TAM}, elektra/p53ER^{TAM} and elektra mice (n=4 per genotype). Mean fluorescence intensity (MFI) of **(c)** CD62L (L-selectin) and **(d)** CD127 (IL7Rα) staining in the CD44^{lo} population of CD4^{+} T-cells from p53ER^{TAM}, elektra/p53ER^{TAM} and elektra mice (n=4 per genotype). *P < 0.001 (two-tailed Student’s t-test). Results are representative of two experiments. Error bars, s.e.

```
CTCCGAAGACTGGATGACTGCCATGGAGGAGTCACAGTCGGATATCAGCCTCGAGCTCCC
||||||||||||||||||||||||||||||||||||||||||||||||||||||||||||
CTCCGAAGACTGGATGACTGCCATGGAGGAGTCACAGTCGGATATCAGCCTCGAGCTCCC

TCTGAGCCAGGAGACATTTTCAGGCTTATGGAAACTACTTCCTCCAGAAGATATCCTGCC
||||||||||||||||||||||||||||||||||||||||||||||||||||||||||||
TCTGAGCCAGGAGACATTTTCAGGCTTATGGAAACTACTTCCTCCAGAAGATATCCTGCC

ATCACCTCACTGCATGGACGATCTGTTGCTGCCCCAGGATGTTGAGGAGTTTTTGAAGG
||||||||||||||||||||||||||||||||||||||||||||||||||||||||||||
ATCACCTCACTGCATGGACGATCTGTTGCTGCCCCAGGATGTTGAGGAGTTTTTGAAGG

CCCAAGTGAAGCCCTCCGAGTGTCAGGAGCTCCTGCAGCACAGGACCCTGTCACCGAGAC
||||||||||||||||||||||||||||||||||||||||||||||||||||||||||||
CCCAAGTGAAGCCCTCCGAGTGTCAGGAGCTCCTGCAGCACAGGACCCTGTCACCGAGAC

CCCTGGGCCAGTGGCCCCTGCCCCAGCCACTCCATGGCCCCTGTCATCTTTTGTCCCTTC
||||||||||||||||||||||||||||||||||||||||||||||||||||||||||||
CCCTGGGCCAGTGGCCCCTGCCCCAGCCACTCCATGGCCCCTGTCATCTTTTGTCCCTTC

TCAAAAAACTTACCAGGGCAACTATGGCTTCCACCTGGGCTTCCTGCAGTCTGGGACAGC
||||||||||||||||||||||||||||||||||||||||||||||||||||||||||||
TCAAAAAACTTACCAGGGCAACTATGGCTTCCACCTGGGCTTCCTGCAGTCTGGGACAGC

CAAGTCTGTTATGTGCACGTACTCTCCTCCCCTCAATAAGCTATTCTGCCAGCTGGCGAA
||||||||||||||||||||||||||||||||||||||||||||||||||||||||||||
CAAGTCTGTTATGTGCACGTACTCTCCTCCCCTCAATAAGCTATTCTGCCAGCTGGCGAA

GACGTGCCCTGTGCAGTTGTGGGTCAGCGCCACACCTCCAGCTGGGAGCCGTGTCCGCGC
||||||||||||||||||||||||||||||||||||||||||||||||||||||||||||
GACGTGCCCTGTGCAGTTGTGGGTCAGCGCCACACCTCCAGCTGGGAGCCGTGTCCGCGC

CATGGCCATCTACAAGAAGTCACAGCACATGACGGAGGTCGTGAGACGCTGCCCCCACCA
||||||||||||||||||||||||||||||||||||||||||||||||||||||||||||
CATGGCCATCTACAAGAAGTCACAGCACATGACGGAGGTCGTGAGACGCTGCCCCCACCA

TGAGCGCTGCTCCGATGGTGATGGCCTGGCTCCTCCCCAGCATCTTATCCGGGTGGAAGG
||||||||||||||||||||||||||||||||||||||||||||||||||||||||||||
TGAGCGCTGCTCCGATGGTGATGGCCTGGCTCCTCCCCAGCATCTTATCCGGGTGGAAGG

AAATTTGTATCCCGAGTATCTGGAAGACAGGCAGACTTTTCGCCACAGCGTGGTGGTACC
||||||||||||||||||||||||||||||||||||||||||||||||||||||||||||
AAATTTGTATCCCGAGTATCTGGAAGACAGGCAGACTTTTCGCCACAGCGTGGTGGTACC

TTATGAGCCACCCGAGGCCGGCTCTGAGTATACCACCATCCACTACAAGTACATGTGTAA
||||||||||||||||||||||||||||||||||||||||||||||||||||||||||||
TTATGAGCCACCCGAGGCCGGCTCTGAGTATACCACCATCCACTACAAGTACATGTGTAA

TAGCTCCTGCATGGGGGGCATGAACCGCCGACCTATCCTTACCATCATCACACTGGAAGA
||||||||||||||||||||||||||||||||||||||||||||||||||||||||||||
```

TAGCTCCTGCATGGGGGGCATGAACCGCCGACCTATCCTTACCATCATCACACTGGAAGA

CTCCAGTGGGAACCTTCTGGGACGGGACAGCTTTGAGGTTCGTGTTTGTGCCTGCCCTGG
||||||||||||||||||||||||||||||||||||||||||||||||||||||||||||
CTCCAGTGGGAACCTTCTGGGACGGGACAGCTTTGAGGTTCGTGTTTGTGCCTGCCCTGG

Supplementary Figure 2: Sequence of p53 in EL4 cell line. Sequence analysis results of the p53 coding sequence from cDNA of B57BL/6J (red font) splenocytes and EL4 lymphoma cell line (black font). Start codon marked in green and STOP codon in yellow.

Primers for Real Time PCR

Gene (mouse)		Sequence
UBC	Forward	CAG CCG TAT ATC TTC CCA GAC T
	Reverse	CTC AGA GGG ATG CCA GTA ATC TA
Mdm2	Forward	TGT GTG AGC TGA GGG AGA TG
	Reverse	CAC TTA CGC CAT CGT CAA GA
Mdmx	Forward	ACA AGA AGA CGG TGG AGG TG
	Reverse	CAC TGC CAC TCA TCC TCA GA
p21	Forward	TCC ACA GCG ATA TCC AGA CA
	Reverse	AGA CAA CGG CAC ACT TTG CT
Cyclin G1	Forward	GCT GGC GCT ATC TAT CCT TG
	Reverse	GGT CAA ATC TCG GCC ACT TA
Bax	Forward	ATG CGT CCA CCA AGA AGC TGA
	Reverse	AGC AAT CAT CCT CTG CAG CTC C
Puma	Forward	CAA GAA GAG CAG CAT CGA CA
	Reverse	CTC CAG GAT CCC TGG GTA AG

Sequencing primers for p53 (mouse)

Gene		Primer
p53	Forward	ATC CTG GCT GTA GGT AGC GA
	Reverse	GGA GGA TTG TGT CTC AGC CC
